# *JAK2*V617F variant allele frequency >50% identifies patients with polycythemia vera at high risk for venous thrombosis

**DOI:** 10.1038/s41408-021-00581-6

**Published:** 2021-12-11

**Authors:** Paola Guglielmelli, Giuseppe G. Loscocco, Carmela Mannarelli, Elena Rossi, Francesco Mannelli, Francesco Ramundo, Giacomo Coltro, Silvia Betti, Chiara Maccari, Sara Ceglie, Patrizia Chiusolo, Chiara Paoli, Tiziano Barbui, Ayalew Tefferi, Valerio De Stefano, Alessandro M. Vannucchi

**Affiliations:** 1 Department of Experimental and Clinical Medicine, CRIMM, Center of Research and Innovation of Myeloproliferative Neoplasms, Azienda Ospedaliero-Universitaria Careggi, University of Florence, Florence, Italy; 2 Section of Hematology, Department of Radiological and Hematological Sciences, Catholic University, Rome, Italy; 3 Fondazione Policlinico Universitario A. Gemelli IRCCS, Rome, Italy; 4 FROM Research Foundation, ASST Papa Giovanni XXIII, Bergamo, Italy; 5 Divisions of Hematology, Mayo Clinic, Rochester, MN USA

**Keywords:** Myeloproliferative disease, Cancer genetics

## Abstract

Arterial (AT) and venous (VT) thrombotic events are the most common complications in patients with polycythemia vera (PV) and are the leading causes of morbidity and mortality. In this regard, the impact of *JAK2*V617F variant allele frequency (VAF) is still debated. The purpose of the current study was to analyze the impact of *JAK2*V617F VAF in the context of other established risk factors for thrombosis in a total of 865 2016 WHO-defined PV patients utilizing two independent cohorts: University of Florence (*n* = 576) as a training cohort and Policlinico Gemelli, Catholic University, Rome (*n* = 289) as a validation cohort. In the training cohort VT free-survival was significantly shorter in the presence of a *JAK2*V617F VAF > 50% (HR 4; *p* < 0.0001), whereas no difference was found for AT (HR 0.9; *p* = 0.8). Multivariable analysis identified *JAK2*V617F VAF > 50% (HR 3.8, *p* = 0.001) and previous VT (HR 2.2; *p* = 0.04) as independent risk factors for future VT whereas diabetes (HR 2.4; *p* = 0.02), hyperlipidemia (HR 2.3; *p* = 0.01) and previous AT (HR 2; *p* = 0.04) were independent risk factors for future AT. Similarly, *JAK2*V617F VAF > 50% (HR 2.4; *p* = 0.01) and previous VT (HR 2.8; *p* = 0.005) were confirmed as independent predictors of future VT in the validation cohort. Impact of *JAK2*V617F VAF > 50% on VT was particularly significant in conventional low-risk patients, both in Florence (HR 10.6, *p* = 0.005) and Rome cohort (HR 4; *p* = 0.02). In conclusion, we identified *JAK2*V617F VAF > 50% as an independent strong predictor of VT, supporting that AT and VT are different entities which might require distinct management.

## Introduction

Polycythemia Vera (PV) is a Philadelphia-negative chronic myeloproliferative neoplasm (MPN) along with essential thrombocythemia, overt myelofibrosis (MF) and pre-fibrotic MF. It is mainly characterized by clonal erythrocytosis associated with an increased risk of thrombo-hemorrhagic complications, progression to MF and, to lesser extent, transformation to acute myeloid leukemia (AML). Other disease features which may negatively impact on quality of life include splenomegaly, systemic and microcirculatory symptoms and pruritus. Almost all patients harbor a somatic *JAK2* (Janus kinase 2; 9p24) mutation, in more than 95% of cases located in exon 14 (*JAK2*V617F), whereas the remaining are heterogeneous *insdel* changes at exon 12 [1]. Current management of PV relies on a two-tiered model that identifies patients at high-risk (age ≥ 60 years and/or history of cardiovascular events (CV)) and low-risk (absence of both risk factors) of thrombosis. In addition to low-dose aspirin and phlebotomies to keep hematocrit below 45%, cytoreductive therapy is recommended for high-risk patients [2, 3]. The International Working Group on Myeloproliferative Neoplasms Research and Treatment (IWG-MRT) identified prior arterial events and hypertension as risk factors for arterial thrombosis whereas prior venous events and age ≥65 years were identified as risk factors for venous thrombosis [4], pointing to arterial and venous thrombosis as two biologically different processes with distinct disease risk factors. More recently, other studies focused on additional risk factors for thrombosis in PV including generic CV risk factors and leukocytosis [5, 6].

Building upon high-throughput sequencing data from collaborative studies between Mayo Clinic, Rochester, USA and University of Florence, Italy, an integrated genetic and clinical survival risk model for PV (MIPSS-PV) was developed, that included *SRSF2* mutations, age >67 years, thrombosis history and leukocytosis (≥15×109/L) as independent risk factors for reduced overall survival, whereas no unique mutation profile was associated with an increased risk for thrombosis [7].

In the 16 years since the discovery of *JAK2*V617F as a driver mutation in MPN, investigators examined the role of *JAK2*V617F as a risk factor either as a qualitative and quantitative variable for thrombosis. Many studies documented a higher risk for thrombotic events in patients with *JAK2*V617F mutated ET or MF compared with negative ones; as a consequence, *JAK2*V617F genotype was incorporated in the International Prognostic Score for Thrombosis in ET [8, 9]. In PV patients, the *JAK2* variant allele frequency (VAF) is highly heterogeneous at diagnosis, with median value around 50%, and correlates with some phenotypic traits; in a previous study a VAF > 75% was associated with higher rate of thrombotic events after diagnosis [10], and a recent study including a large cohort of Chinese patients reported a 4.6 times higher incidence of thrombosis in PV patients with *JAK2*V617F VAF ≥ 50% [11]. Moreover, a *JAK2*V617F VAF > 50% was also associated with increased fibrotic transformation [12]. At the light of recent observations, it seems that not only circulating blood cells, but also the vessels can participate in thrombosis. Noteworthy, the *JAK2*V617F mutation has been reported in endothelial cells in some MPN patients, especially those with thrombosis [13, 14], raising the question of their thrombogenicity. Moreover, it is still unclear why splanchnic vein thrombosis (SVT), including hepatic veins, portal veins, splenic veins, or mesenteric veins are a privileged site of thrombosis during MPN, particularly in PV patients, and it raises the hypothesis of a specific physiopathology. SVT are more common in young female patients and in those with a low *JAK2*V617F allele burden [15–17], whereas in a recent paper a *JAK2*-mutant allele burden ≥50% and the presence of chromatin/spliceosome/*TP53* mutations identified high-risk SVT-MPN patients with a worse event-free (including both AML and MF progression) and overall survival at 10 years [18].

However, whether the *JAK2*V617F VAF is prognostically meaningful, particularly concerning thrombosis risk in PV patients, is still debated. The aim of the current study was to evaluate the impact of *JAK2*V617F VAF at diagnosis on rate of arterial and venous thrombosis (with the exclusion of SVT from the latter), in the context of other established risk factors, in 2016 WHO-defined PV patients belong to a training cohort collected from Center of Research and Innovation in Myeloproliferative Neoplasms (CRIMM), University of Florence, Italy and a validation cohort from Policlinico Gemelli, Catholic University, Rome, Italy.

## Materials and methods

### Study population

This retrospective study was approved by the Institutional review board by the Local Ethics Committee at University of Florence and Policlinico Gemelli, Catholic University, Rome. The study was conducted in accordance with the ethical guidelines of the Declaration of Helsinki. Study patients were selected from respective institutional databases provided they were confirmed according to the 2016 WHO criteria for PV diagnosis and written, informed consent was obtained from all living subjects; treatment approaches were in accordance with standard of care at the time of initial diagnosis. Diagnostic criteria for leukemic and myelofibrotic transformation were revised according to the 2016 WHO classification [19] and international working group for MPN research and treatment (IWG-MRT) criteria [20], respectively. *JAK2* exon 12 mutated PV patients were excluded, and all patients were annotated for *JAK2*V617F VAF, determined within 3 years from diagnosis, according to previous published methods [21]. Bone marrow biopsies were internally reviewed to adhere to current criteria and in those without an evaluable bone marrow histology, PV diagnosis relied on the presence of both elevated hemoglobin levels (i.e., >16.5/16.0 g/dl in males and females, respectively), presence of *JAK2*V617F mutation and low serum erythropoietin, according to the latest WHO criteria. Arterial thrombosis included ischemic stroke, transient ischemic attack, retinal artery occlusion, coronary arterial disease, and peripheral arterial disease, whereas venous thrombosis included cerebral venous thrombosis, deep vein thrombosis of the limbs, pulmonary embolism and superficial vein thrombosis. All reported thrombotic events were objectively identified by imaging, serological biomarkers and/or electrocardiogram. Thrombotic events were considered as post diagnosis if occurring at least 4 weeks after PV diagnosis whereas thrombotic events before diagnosis included all events that occurred at any time prior to diagnosis. Microcirculatory symptoms included dizziness, headaches, visual disturbances, erythromelalgia, distal paresthesia and acrocyanosis. Major bleedings were defined based on International Society on Thrombosis and Hemostasis (ISTH) [22] definition as: gastrointestinal, internal organ, intraarticular, cerebrovascular, retroperitoneal bleed or any bleeding requiring medical and/or surgical intervention, hospitalization and/or resulting in death.

### Statistical methods

For the purposes of the current study, only the first arterial and venous event occurring after PV diagnosis was considered. Continuous variables were summarized as median and minimum-maximum ranges. Distribution of continuous variables was compared using nonparametric test (Mann–Whitney), while nominal variables were compared with the Chi-square test. Cox proportional hazard regression model was used for univariate and multivariable analysis for arterial and venous thrombosis-free survival. Survival analysis was considered from the date of diagnosis to date of death or last contact. Thrombosis-free, leukemia-free and myelofibrosis-free survival calculations considered the event as the uncensored variable. The Kaplan–Meier method was used to construct time-to-event curves, which were compared by log-rank test, whereas a receiver operating characteristics curve (ROC) was used to determine the best thresholds of VAF using area under curve (AUC) estimate through Youden index analysis. For all tested hypotheses, two-tailed p values less than 0.05 were considered significant. All the statistical analyses were performed with SPSS software, version 27 (IBM-Corp), JMP Pro 15.1.0 software from SAS Institute (Cary, NC) and Statistical Package R version 4.1.1.

## Results

### Patient characteristics of the training cohort

Among a total of 576 patients from the University of Florence training cohort (seen 1981-2020), median age at diagnosis was 61.4 years (range, 18–92) and 58.2% were male; 60.4% were at high-risk for thrombosis based on current risk stratification. Median *JAK2*V617F VAF was 41.5% (range, 0.3–100). Leukocytosis (≥11×10^9^/L) was documented in 37.9% of patients, whereas palpable splenomegaly, microcirculatory symptoms, constitutional symptoms, and pruritus were reported in 35.7% (*n* = 194), 31.2% (*n* = 170), 12.2% (*n* = 69) and 39.5% (*n* = 223) of patients, respectively. The incidence of cardiovascular (CV) risk factors at diagnosis was as follows: hypertension (56%), diabetes (10.3%), hyperlipidemia (15.9%), and active smoking (16%). With a median survival of 21.7 years, 13% and 2.1% of patients experienced myelofibrotic and leukemic transformation during the course of disease, respectively; 16.7% of patients died. Clinical and laboratory patients’ characteristics are detailed in Table 1.

**Table 1:** Laboratory and clinical characteristics of *JAK*2V617F positive PV patients from training cohort stratified by their variant allele frequency (VAF > / ≤ 50%).

Laboratory and clinical characteristics	All patients (*n* = 576)	*JAK2* V617F VAF ≤ 50% (n = 369; 64.1%)	*JAK2* V617F VAF > 50 % (n = 207; 35.9%)	*P* values*
Age in year; median (range)	61.4 (18–92)	62.2 (18–92)	59.4 (22–91)	0.2
Age ≥ 60 years; *n* (%)	311 (54)	210 (56.9)	101 (48.8)	0.06
Sex females; *n* (%)	241 (41.8)	146 (39.6)	95 (45.9)	0.1
Risk stratification; high risk *n* (%)	348 (60.4)	235 (63.7%)	113 (54.6%)	**0.04**
White blood cells x 10^9^/L; median (range) N evaluable=530	9.8 (4.5–38.5)	9.2 (4.5–38.5)	12.1 (5.3–34.1)	**<0.0001**
White blood cells ≥ 11 ×10^9^/L; *n* (%) N evaluable=530	201 (37.9)	96 (27.3)	105 (59)	**<0.0001**
Hemoglobin, g/dL; median (range)	17.8 (15.8–24.5)	17.6 (15.8–22.4)	18.3 (15.9–24.5)	**<0.0001**
Hematocrit, % median (range)	53.7 (47.9–75.9)	52.7 (47.9–70)	56.2 (48.5–75.9)	**<0.0001**
Platelets x 10^9^/L; median (IQR) N evaluable =527	458 (154–1638)	493 (154–1638)	402 (155–1200)	**<0.0001**
Lactate dehydrogenase UI/L; median (range) N evaluable =428	278.5 (123–678)	260 (123–675)	352 (169–678)	**<0.0001**
Palpable splenomegaly; *n* (%) N evaluable =543	194 (35.7)	92 (26.2)	102 (53.1)	**<0.0001**
Constitutional symptoms; *n* (%) N evaluable =564	69 (12.2)	38 (10.6)	31 (15.2)	0.1
Pruritus; *n* (%) N evaluable =565	223 (39.5)	110 (30.5)	113 (55.4)	**<0.0001**
Arterial thrombosis before/at diagnosis; *n* (%)	76 (13.2)	51 (13.8)	25 (12.1)	0.5
Arterial thrombosis at follow-up; *n* (%)	49 (8.5)	27 (7.3)	22 (10.6)	0.2
Venous thrombosis before/at diagnosis; *n* (%)	52 (9)	28 (7.6)	24 (11.6)	0.1
Venous thrombosis at follow-up; *n* (%)	39 (6.8)	9 (2.4)	30 (14.5)	**<0.0001**
Major bleeding before/at diagnosis; *n* (%)	20 (3.5)	14 (3.8)	6 (2.9)	0.6
Major bleeding at follow-up; *n* (%)	34 (5.9)	17 (4.6)	17 (8.2)	0.08
Cardiovascular risk factors (at least one); *n* (%) N evaluable=476	295 (61)	198 (65.3)	97 (56.1)	0.05
Active smocking; *n* (%) N evaluable=430	69 (16)	57 (20.4)	12 (7.9)	**0.0004**
Diabetes; *n* (%) N evaluable=438	45 (10.3)	27 (9.5)	18 (11.6)	0.5
Hyperlypidemia; *n* (%) N evaluable=439	70 (15.9)	51 (18)	19 (12.3)	0.1
Hypertension; *n* (%) N evaluable=446	250 (56)	161 (55.7)	89 (56.7)	0.8
Microcirculatory symptoms; *n* (%) N evaluable=545	170 (31.2)	100 (28.7)	70 (35.5)	0.1
MF progression; *n* (%)	75 (13)	16 (4.3)	59 (28.5)	**<0.0001**
AML progression: *n* (%)	12 (2.1)	4 (1.1)	8 (3.9)	**0.03**
Death; *n* (%)	96 (16.7)	37 (10)	59 (28)	**<0.0001**
Median survival (years)	21.7	23.9	19.6	0.5

* Significant *p* values highlighted in bold refer to comparison of VAF ≤ 50% and >50%.

A total of 76 (13.2%) patients had an arterial thrombotic event before or coincident with PV diagnosis, whereas 49 (8.5%) patients had at least one arterial event during follow-up. As regards venous thrombotic events, 52 (9%) and 39 (6.8%) patients had a venous thrombotic event before/at or after PV diagnosis, respectively. Overall a total of 88 thrombotic events occurred in 78 patients (13.5%), 1.7% of whom having experienced both an arterial and venous thrombosis. Arterial events occurred before/at PV diagnosis were mainly represented by cerebrovascular and cardiovascular events. Ischemic stroke, transient ischemic attack and acute myocardial infarction occurred in 22 (28.9%), 17 (22.4%) and 26 (34.2%) patients, respectively. Similarly, the most common arterial thrombosis during follow-up were transient ischemic attack (*n* = 15; 30.6%), followed by acute myocardial infarction (*n* = 13; 26.5%) and ischemic stroke (*n* = 7; 18%). Concerning venous events, deep vein thrombosis, superficial vein thrombosis and pulmonary embolism, represented the most common events both before/at (53.8%, 17.3% and 15.4%, respectively) and after (48.7%, 20.5% and 17.9%, respectively) diagnosis. Overall, the incidence rate of thrombosis was 0.83%/year for VT and 1.04%/year for AT. In Table 2 the types of arterial and venous thrombosis occurred before/at or after PV diagnosis are detailed.

**Table 2:** Type of thrombotic events occurring before/at diagnosis or during follow-up in training cohort.

University of Florence cohort (*n* = 576)		
	Before/at diagnosis	During follow-up
**Arterial thrombosis (*n*, %)**	*n* = 76	*n* = 49
Acute myocardial infarction	26 (34.2)	13 (26.5)
Unstable angina	4 (5.3)	5 (10.2)
Stroke	22 (28.9)	12 (24.5)
Transient ischemic attack	17 (22.4)	15 (30.6)
Peripheral arterial thrombosis	5 (6.6)	4 (8.2)
Abdominal thrombosis	1 (1.3)	–
Retinal thrombosis	1 (1.3)	–
**Venous thrombosis** **(*n*, %)**	*n* = 52	*n* = 39
Deep vein thrombosis	28 (53.8)	19 (48.7)
Pulmonary embolism	8 (15.4)	7 (17.9)
Pulmonary embolism + deep vein thrombosis	1 (1.9)	2 (5.1)
Cerebral vein thrombosis	3 (5.8)	1 (2.6)
Superficial vein thrombosis	9 (17.3)	8 (20.5)
Retinal thrombosis	3 (5.8)	2 (5.1)

### Risk factors for thrombosis in training cohort

**Fig. 1:**
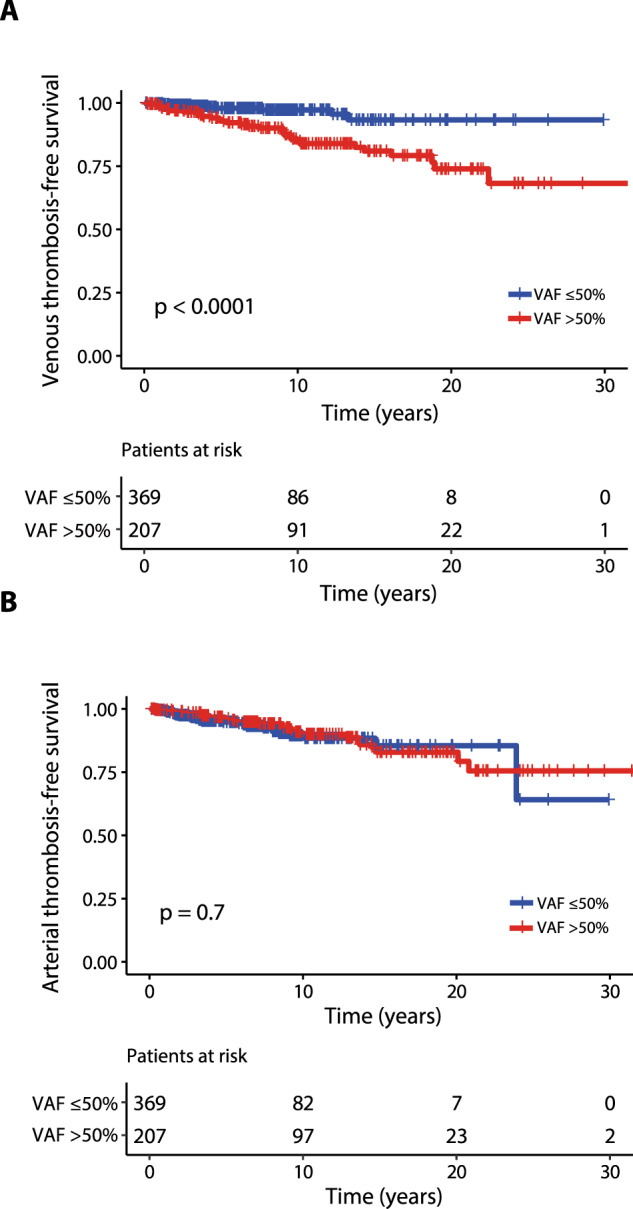
Venous and arterial thrombosis-free survival for *JAK2*V617F positive PV patients from training cohort stratified by their *JAK2*V617F VAF (>50% vs ≤50%). Kaplan–Meier curves representing venous thrombosis-free survival (**A**) and arterial thrombosis-free survival (**B**) including a total of 576 PV patients. The number of patients at risk for each time point is shown below the graph. Tick marks indicate censored data.

Considering the patients included in the Florence cohort we found that *JAK2*V617F VAF as a continuous variable was correlated with the risk of venous thrombosis after diagnosis (*p* = 0.003; HR 1; 95% CI 1–1.1) but not with arterial thrombosis (*p* = 0.8; HR 1; 95% CI 0.9–1). A receiver operating characteristic (ROC) curve was used to determine the best cut-off level for *JAK2*V617F VAF predicting venous thrombosis; the curve showed an area under curve (AUC) of 0.72 (Supplementary Fig. 1), and the best cut-off value was VAF = 50%. Accordingly, we divided PV patients in those with VAF ≤ 50% (369; 64.1%) and >50% (207; 35.9%). Compared to patients with *JAK2*V617F VAF ≤ 50%, those with VAF > 50% displayed higher white blood cells count (*p* < 0.0001), higher hematocrit and hemoglobin levels (*p* < 0.0001 each), lower platelet count (*p* < 0.0001), had more commonly palpable splenomegaly (*p* < 0.0001) and pruritus (*p* < 0.0001). A comparison of laboratory and clinical variables in patients stratified by their VAF (> vs ≤ 50%) is outlined in Table 1. The rate of venous thrombosis during follow-up was significantly higher in the presence of a *JAK2*V617F VAF > 50% (2.4% vs 14.5%; *p* < 0.0001), that was associated with a significantly worse thrombosis free survival HR 4, 95% CI 1.9-8.6, *p* < 0.0001; (Fig. 1A). Conversely, no difference was found for arterial thrombosis (HR 0.9, 95% CI 0.5-1.6, *p* = 0.7; Fig. 1B). In addition to VAF > 50%, univariate analysis for venous thrombosis-free survival (V-TFS) identified a previous venous thrombosis (HR 2.9, 95% CI 1.4–6.1; *p* = 0.006), leukocytosis ≥11 ×109/L (HR 1.9; 95% CI 1.1–3.4; *p* = 0.02) and palpable splenomegaly (HR 1.9, 95% CI 1-3.6; *p* = 0.04) as risk factors for venous thrombosis. Age ≥60 years was not significant (HR 1, 95% CI 0.9-1; *p* = 0.9). Multivariable analysis confirmed VAF > 50% (HR 3.8, 1.7–8.6; *p* = 0.001) and previous venous thrombosis (HR 2.2, 95% CI 1.1-5; *p* = 0.04) as independent risk factors for future venous thrombosis. In contrast, univariate analysis for arterial thrombosis-free survival (A-TFS) identified history of arterial thrombosis (HR 2.5, 95% CI 1.3–4.9; *p* = 0.007), diabetes (HR 3.3; 95% CI 1.6–6.5; *p* = 0.0007), hyperlipidemia (HR 3.1, 95% CI 1.7-5.6; *p* = 0.0003) and hypertension (HR 2, 95% CI 1–3.9; *p* = 0.04) as predictors of a subsequent arterial event; age ≥60 years showed only a trend for significance (HR 1.7; 95% CI 0.9–3.1, *p* = 0.08). Multivariable analysis for A-TFS identified diabetes (HR 2.4; 95% CI 1.2–5, *p* = 0.02), hyperlipidemia (HR 2.3; 9% CI 1.2–4.3, *p* = 0.01) and previous arterial event (HR 2.1; 95% CI 1–4.2, *p* = 0.04) as independent predictors of a subsequent arterial event. Univariate and multivariable analysis, considering clinical and laboratory variables, for both venous and arterial thrombosis-free survival among patients from the Florence cohort are reported in Table 3.

**Table 3:** Univariate and multivariable analysis for venous and arterial thrombosis-free survival among PV patients from training cohort.

Clinical and laboratory variables	Venous thrombosis-free survival		Arterial thrombosis-free survival	
	Univariate analysis HR (95% CI) P*	Multivariable analysis HR (95% CI) P*	Univariate analysis HR (95% CI) P*	Multivariable analysis HR (95% CI) P*
Age in years	1 (0.9–1); 0.6		1 (1–1.1); **0.04**	1 (1-1.1); 0.3
Age ≥ 60 years	1 (0.5–1.9); 0.9		1.7 (0.9–3.1); 0.08	
Male sex	0.7 (0.4–1.4); 0.3		1.4 (0.8–2.5); 0.2	
High-risk (conventional risk stratification)	1.6 (0.8–3.2); 0.1		1.6 (0.9–2.9); 0.1	
White blood cells (x 10^9^/L)	1 (1–1.1); 0.1		1 (0.9–1); *p* = 0.5	
White blood cells (≥ 11 ×109)	1.9 (1.1–3.4); **0.02**	0.9 (0.5–1.9); 0.9	0.8 (0.5–1.5); 0.5	
Hemoglobin (g/dL)	1 (0.8–1.3); 0.7		0.9 (0.7–1.1); 0.3	
Hematocrit (%)	1 (0.61–1.1); 0.6		1 (0.9–1); 0.4	
Platelet count (x 10^9^/L)	1 (0.9–1); 0.1		1 (0.9–1); 0.7	
Lactate dehydrogenase (UI/L)	1 (0.9–1); 0.6		1 (1–1.1); 0.09	
*JAK2* V617F VAF (%) (continuous variable)	1 (1–1.1); **0.003**		1 (0.9–1); 0.9	
*JAK2* V617F VAF > 50%	4 (1.9–8.6); **0.0003**	3.8 (1.7–8.6); **0.001**	0.9 (0.5–1.6); 0.7	
Palpable splenomegaly	1.9 (1–3.6); **0.04**	1.3 (0.7–2.7); 0.3	1 (0.6–1.9); 0.8	
Arterial thrombosis before/at diagnosis	1.7 (0.8–1.4); 0.2		2.5 (1.3–4.9); **0.007**	2.1 (1–4.1); **0.04**
Venous thrombosis before/at diagnosis	2.9 (1.4–6.1); **0.006**	2.2 (1.1–5); **0.04**	0.5 (0.2–1.7); 0.3	
Major bleeding before/at diagnosis	0.05 (1–460); 0.5		0.05 (0.1–120); 0.5	
Diabetes	1.4 (0.5–4.1); 0.5		3.3 (1.6–6.5); **0.0007**	2.4 (1.1–4.9); **0.02**
Hypertension	0.9 (0.4–1.8); 0.7		2 (1–3.9); **0.03**	1.4 (0.7–2.8); 0.3
Hyperlipidemia	1.7 (0.7–3.9); 0.2		3.1 (1.7–5.6); **0.0003**	2.3 (1.2–4.3); **0.01**
Active smoking	1.4 (0.5–3.6); 0.5		0.7 (0.3–2.1); 0.6	

*Significant *p* values are highlighted in bold.

*PV* polycythemia vera, *HR h*azard ratio, *VAF* variant allele frequency, *CI* confidence interval.

**Fig. 2:**
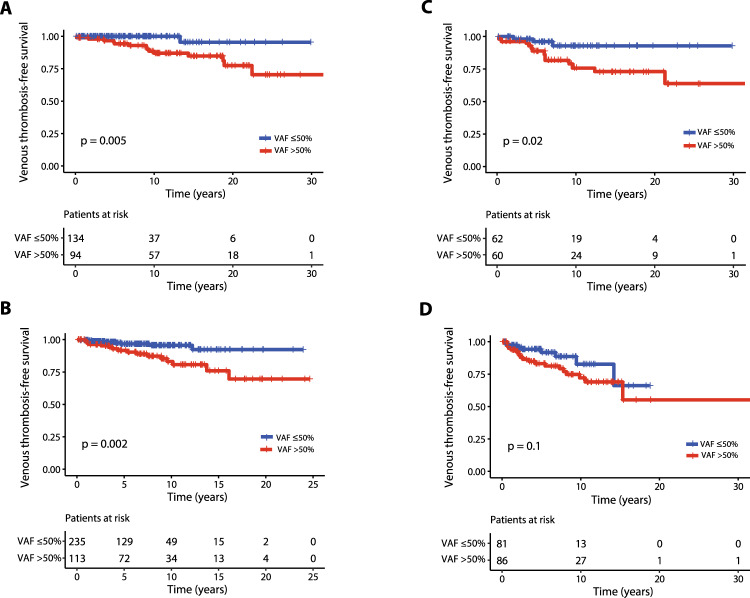
Venous thrombosis-free survival for low and high-risk *JAK2*V617F positive PV patients from training and validation cohorts stratified by their *JAK2*V167F VAF (>50% vs ≤50%). Kaplan–Meier curves representing venous thrombosis-free survival for training cohort considering those at low risk (**A**) and high risk (**B**) at diagnosis. The same analysis on validation cohort patients at low-risk (**C**) and high-risk (**D**). The number of patients at risk for each time point is shown below the graph. Tick marks indicate censored data.

Finally, in order to mitigate the confounding effect of different therapeutic approach concerning the impact of VAF > 50% on V-TFS, patients were stratified according to the conventional risk category attributed at diagnosis, which correlated with cytoreductive therapy (high risk, >90% cases received hydroxyurea) or phlebotomy only (in the majority of low risk patients) along with low-dose acetylsalicylic acid (ASA); the Kaplan-Meier curves presented in Fig. 2A and B show that a *JAK2*V617F VAF > 50% remains significant for the risk of future venous events both in low-risk (HR 10.6; 95% CI 1.4-81.5, *p* = 0.005) and high-risk patients (HR 3.5; 95% CI 1.5–8.3, *p* = 0.002), respectively. Moreover, patients with VAF > 50% showed a higher risk for MF progression (HR 3.6; 95% CI 2–6.3; *p* < 0.0001) without any differences in leukemic progression (HR 1.1; 95% CI 0.3–3.7; *p* = 0.9) and overall survival (HR 1.1; 95% CI 0.7–1.8; *p* = 0.5), Supplementary Fig. 2A–C.

### Patient characteristics of the validation cohort

In the Policlinico Gemelli, Catholic University database a totally of 289 PV patients, with confirmed 2016 WHO criteria, who were fully annotated for *JAK2*V617F VAF were included in the current analysis.

Median age at diagnosis was 61 years (range, 19–89) and 49.5% were male; 57.8% were at high-risk for thrombosis based on current risk stratification. Median *JAK2* VAF was 51% (range, 1–100). Leukocytosis (≥11×10^9^/L) was documented in 35.1% of patients, whereas palpable splenomegaly, microcirculatory symptoms, constitutional symptoms, and pruritus were reported in 35.1% (*n* = 97), 38.4% (*n* = 111), 11.7% (*n* = 33) and 38.9% (*n* = 110) of patients, respectively.

Among CV risk factors, hypertension, diabetes, hyperlipidemia, and active smoking were reported in 47.4%, 5.9%, 14.9% and 17.6% of patients, respectively. Median survival of the whole patients’ cohort was not reached and during the course of disease, 7.9% and 1.7% of the patients experienced myelofibrotic and leukemic progression, respectively; 6.6% of patients died. Clinical and laboratory patients’ characteristics of the Rome cohort are detailed in Table 4.

**Table 4:** Laboratory and clinical characteristics of *JAK*2V617F positive PV patients from validation cohort stratified by their variant allele frequency (VAF > / ≤ 50%).

Laboratory and clinical characteristics	All patients (*n* = 289)	*JAK2* V617F VAF ≤ 50% (*n* = 143; 49.5%)	*JAK2* V617F VAF > 50 % (*n* = 146; 50.5%)	*P* values*
Age in year; median (range)	61 (19–89)	60 (21–89)	62 (19–89)	0.67
Age ≥ 60 years; *n* (%)	150 (51.9)	72 (50.3)	78 (53.4)	0.60
Sex females; *n* (%)	146 (50.5)	68 (47.6)	78 (53.4)	0.31
Risk stratification; high risk *n* (%)	167 (57.8)	81 (56.6)	86 (58.9)	0.69
White blood cells x 10^9^/L; median (range) N evaluable=245	10.05 (1.0–25.2)	9.6 (5.3–22.8)	10.2 (1.0–25.2)	**0.02**
White blood cells ≥ 11 ×10^9^/L; *n* (%) N evaluable=245	86 (35.1)	38 (30.2)	48 (40.3)	0.09
Hemoglobin, g/dL; median (range)	17.1 (12.1–24.0)	16.8 (13.9–21.2)	17.4 (12.1–24.0)	**0.0085**
Hematocrit, % median (range)	52.9 (40.6–71.0)	51.6 (41.5–66.0)	54.4 (40.6–71.0)	**0.0001**
Platelets x 10^9^/L; median (IQR) N evaluable =251	503 (368.5–647.0)	550 (412.0–695.7)	443 (334.0–599.0)	**0.0009**
Palpable splenomegaly; *n* (%) N evaluable =276	97 (35.1)	39 (27.3)	58 (42.3)	**0.013**
Constitutional symptoms; *n* (%) N evaluable =283	33 (11.7)	14 (9.9)	19 (13.5)	**0.03**
Pruritus; *n* (%) N evaluable =283	110 (38.9)	48 (33.8)	62 (44.0)	0.08
Arterial thrombosis before/at diagnosis; *n* (%)	50 (17.3)	27 (18.9)	23 (15.7)	0.48
Arterial thrombosis at follow-up; *n* (%)	45 (15.6)	20 (14.0)	25 (17.1)	0.46
Venous thrombosis before/at diagnosis; *n* (%)	36 (12.5)	17 (11.9)	19 (13.0)	0.77
Venous thrombosis at follow-up; *n* (%)	43 (14.9)	11 (7.7)	32 (21.9)	**0.0006**
Major bleeding at follow-up; *n* (%)	32 (11.1)	15 (10.5)	17 (11.6)	0.75
Cardiovascular risk factors; *n* (%)	184 (63.7)	97 (67.8)	87 (59.6)	0.14
Microcirculatory symptoms; *n* (%)	111 (38.4)	58 (40.6)	53 (36.3)	0.46
MF progression; *n* (%)	23 (7.9)	4 (2.8)	19 (13.0)	**0.0013**
AML progression: *n* (%)	5 (1.7)	2 (1.4)	3 (2.1)	0.67
Death; *n* (%)	19 (6.6)	4 (2.8)	15 (10.3)	**0.0028**
Median survival (years)	Not reached	Not reached	25.7	0.13

*significant *p* values highlighted in bold refer to comparison of ≤50% and >50%.

In the validation cohort, the incidence of arterial thrombosis was 17.3% (*n* = 50) before/at diagnosis and 15.6% (*n* = 45) during follow-up. The most common arterial events were represented by cerebrovascular thrombosis (57.9% and 58.8% at diagnosis and during follow-up, respectively) followed by cardiovascular events (39.5% and 26.8% at diagnosis and during follow-up, respectively). Regarding venous thrombosis, 36 events (incidence of 12.5%) were reported before/at diagnosis and 43 events (incidence of 14.9%) during follow-up. Deep vein thrombosis and pulmonary embolisms were more common at diagnosis (44.5% and 22.2%), whereas superficial vein thrombosis were more common during follow-up (46.5%). Overall, the incidence rate of thrombosis was 1.92%/year for VT and 2.08%/year for AT.

The types of arterial and venous events occurred both at diagnosis (data on type of events occurred before diagnosis were not available) or during follow-up in validation cohort are outlined in Supplementary Table 1.

### Validation of risk factors for thrombosis

**Fig. 3:**
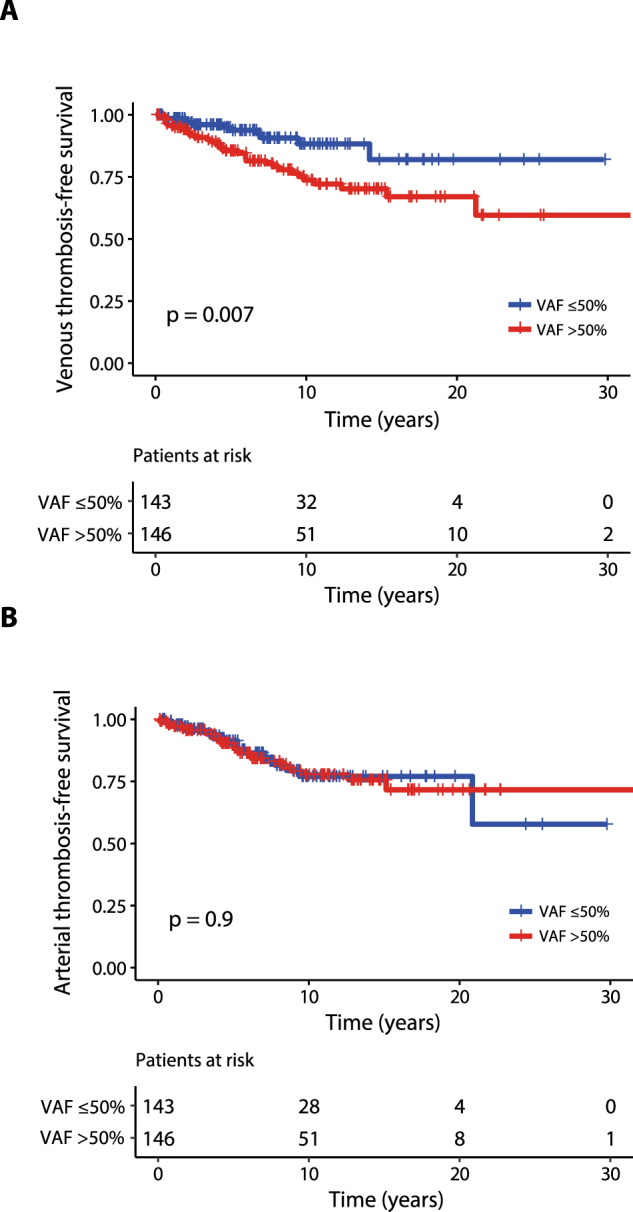
Venous and arterial thrombosis-free survival for *JAK2*V617F positive PV patients from validation cohort stratified by their *JAK2*V617F VAF (>50% vs ≤50%). Kaplan–Meier curves representing venous thrombosis-free survival (**A**) and arterial thrombosis-free survival (**B**) including a total of 289 PV patients. The number of patients at risk for each time point is shown below the graph. Tick marks indicate censored data.

Confirming findings in the training cohort, the rate of venous thrombosis during follow-up was significantly higher in the presence of a *JAK2*V617F VAF > 50% (HR 2.9, 95% CI 1.2–4.2; *p* = 0.007); (Fig. 3A), whereas no difference was found for arterial thrombosis (HR 1, 95% CI 0.6-1.9, *p* = 0.9; Fig. 3B). A comparison of laboratory and clinical variables stratifying patients by their VAF (>50% vs ≤50%) is detailed in Table 4. In addition to VAF > 50%, univariate analysis for V-TFS identified previous venous thrombosis (HR 5.1, 95% CI 1.8–14.2; *p* = 0.002) as risk factors for future venous thrombosis. Multivariable analysis confirmed *JAK2*V617F VAF > 50% (HR 2.4, 95% CI 1.2–4.8; *p* = 0.01) and previous venous thrombosis (HR 2.8, 95% CI 1.4–5.7; *p* = 0.005) as independent risk factors for future venous events. Regarding A-TFS, univariate analysis identified history of arterial thrombosis as a quite significant predictor of future arterial event (HR 2.4; 95% CI 0.9–6; *p* = 0.07). Conversely, age ≥60 years (HR 1.1; 95% CI 0.6–2; *p* = 0.7) and presence of CV risk factors at diagnosis (HR 0.8; 95% CI 0.5–1.5; *p* = 0.6) were not significant. Finally, *JAK2*V617F VAF > 50% significantly affected V-TFS in low-risk patients (HR 4; 95% CI 1.1–13.9, *p* = 0.02, Fig. 2C) whereas the impact of VAF > 50% was close to significance considering high-risk patients’ cohort (HR 1.9; 95% CI 0.8–4.4, *p* = 0.1, Fig. 2D).

## Discussion

Cardiovascular and thromboembolic events are the most relevant complications in patients with PV and are the major cause of morbidity and mortality. Nowadays, current treatment strategies in PV are directed at preventing thrombotic complications. In this regard, two risk categories are conventionally considered: high (age ≥ 60 years or thrombosis history) and low (absence of both risk factors). All patients require phlebotomy to keep hematocrit below 45% and once-daily low-dose ASA, whereas cytoreductive therapy is recommended for high-risk patients. However, despite adherence to treatments, thrombotic risk remains significant at varying degrees during follow-up [23]. A retrospective analysis of patients with MPNs from the Swedish Cancer Registry (*n* = 9429; PV, *n* = 3001), including patients followed from 1987 to 2009, reported that at 3 months after diagnosis, patients with PV had an approximately 3- and 9.7-fold higher risk of arterial thrombosis and venous thrombosis, respectively, compared to controls matched for age and sex [24]. Epidemiological data concerning incidence of arterial and venous thrombosis before/at diagnosis in PV patients derive from two seminal studies. In the European Collaboration on Low-Dose Aspirin in Polycythemia Vera (ECLAP) study arterial and venous thrombosis history before/at diagnosis was documented in 27% and 11% of patients, respectively [2]. A significant lower incidence for arterial thrombosis was documented in CYTO-PV trial (arterial 17%/venous 12%) that was conducted several years later [25]. Similar results (arterial 16%/venous 7.4%) were reported in a study including 1545 contemporary PV patients followed in 7 centers in Italy, Austria, and the United States referring to the International Working Group for Myeloproliferative Neoplasms [4]. Prospective and retrospective studies investigated the prognostic role of risk factors for thrombosis in large cohorts of PV patients. However, few data focused on including the prognostic contribution of *JAK2*V617F VAF on thrombosis in PV patients are available and the role of VAF is still uncertain.

The aim of the current study was to specifically evaluate in 2016 WHO-defined PV patients the impact of *JAK2*V617F VAF on rate of arterial and venous thrombosis, excluding SVT, in the context of other established risk factors. First, a training cohort from University of Florence was examined. *JAK2*V617F VAF as a continue variable was correlated with the risk of venous thrombosis after diagnosis (*p* = 0.003) but not with arterial thrombosis (*p* = 0.8). Multivariable analysis identified *JAK2*V617F VAF > 50% (HR 3.8, *p* = 0.001) and previous VT (HR 2.2; *p* = 0.04) as independent risk factors for future VT whereas diabetes (HR 2.4; *p* = 0.02), hyperlipidemia (HR 2.3; *p* = 0.01) and previous AT (HR 2; *p* = 0.04) were independent risk factors for future AT. *JAK2*V617F VAF > 50% (HR 2.4; *p* = 0.01) and previous VT (HR 2.8; *p* = 0.005) were confirmed as independent predictors of future VT also in the validation cohort including 289 PV patients from Policlinico Gemelli, Catholic University, Rome. In both cohorts, we confirmed the reported impact of previous venous thrombosis for subsequent venous events and previous arterial thrombosis for arterial events [4, 6]; CV risk factors were significant risk factors for AT in training cohort but not confirmed in validation cohort. Conversely, age was not significant in either cohorts, possibly reflecting changing epidemiology of the disease as well as improvement of general health status and longer life expectation, compared to older studies. From an epidemiological point of view, in the current study including a total of 865 patients, the cumulative incidences of arterial and venous thrombosis before/at diagnosis was 14.6% and 10.2% respectively, similarly to the most recent trials reported above [4, 25]. Considering thrombotic events after diagnosis, the incidence rate was 0.83%/year for VT and 1.04%/year for AT in Florence cohort whereas rates were higher in Rome cohort: 1.92%/year for VT and 2,08%/year for AT. Pooled incidence rates were 1.36%/year for AT e 1.18%/year for VT. Altogether, the frequency of global thrombotic events in our study (2.4%/years) was slightly lower than those reported in the Cyto-PV trial (2.7%/years) [26]. Earlier diagnosis and treatment, including PV patients fully diagnosed according to the latest 2016 WHO classification, improved management of CV factors and a more appropriate use of phlebotomy, antiplatelet and cytoreductive drugs, might reflect a change of clinical epidemiology of thrombotic events occurring after diagnosis. This notwithstanding, thrombotic risk remains substantial, particularly in those defined at low risk. At this regard, the LOW-PV trial (NCT03003325) is ongoing and evaluating whether the addition of ropeginterferon alfa-2b to phlebotomy and antiplatelet therapy could improve the outcomes. Interim analysis results documented that ropeginterferon alfa-2b was superior in maintaining the recommended hematocrit value of less than 45% with a deepest mean change of *JAK2*V617F VAF at 12-months follow-up (−10.43% vs −1.03%; *p* = 0.006) [27]. More complete and mature data will help to understand how the impact of the reduction of *JAK2*V617F, if confirmed, may have prognostic implications, in particular on thrombotic risk, in these low-risk patients [28, 29].

Recent advances in our understanding of the roles of clonal hematopoiesis, *JAK2*V617F mutation, endothelial cells and inflammation in thrombosis risk are providing new insights into pathophysiology of PV [30, 31]. In a recent study, RNA sequencing in granulocytes of PV patients documented higher expression levels of *F3*, *SELP*, *VEGFA*, and *SLC2A1*, that were directly correlated with *JAK2* expression and *JAK2*V617F allele burden in patients with a history of thrombosis [32].

The main potential limitation of our study concerns its retrospective design; moreover, we did not collect data on the use of anticoagulants, dose of antiplatelet therapy, differences in the use of cytoreductive drugs, statins and hypertension treatments along with concomitant thrombophilic disorders, which could confound associations with thrombosis. The intrinsic potential drawbacks of the retrospective design could explain the differences in the incidence rate of thrombosis between the training and validation cohort. However, the study has also strengths regarding the analysis of two large and independent cohorts from Centers with experience in MPNs, the well-defined patient population including a total of 865 PV patients diagnosed according to the latest 2016 WHO classification criteria and managed according to the best-practice principles, the evaluation of patients at diagnosis, and the long follow-up period. Furthermore, due to the low-rate of events and the long follow-up required, it is unlikely that prospective studies with this primary endpoint might be conducted. In summary, the present study indicates that PV patients with a *JAK2*V617F VAF > 50% suffer from increased rate of venous events over time, particularly in conventionally defined low-risk patients. On the other hand, *JAK2*V617F VAF > 50% has no impact on arterial thrombosis. Moreover, it confirms the importance of a history of thrombosis in predicting future thrombotic events and supports that arterial and venous events are distinct entities with specific risk factors, including *JAK2*V617F VAF, that require careful recognition and management. In conclusion, this study does not aim to develop a new global thrombotic risk model for PV patients since *JAK2*V617F VAF correlated with VT but not with AT. However, these findings suggest the classical thrombotic risk model based on age and previous thrombosis may not be enough informative to tailor adequate treatment, especially in the setting of VT prevention. Therefore, in parallel to conventional risk stratification, *JAK2*V617F VAF might receive consideration for an individualized risk assessment. Whether this should result in modification of the current treatment approach is beyond the scope of this work and would require prospective evaluation.

## Supplementary information

Supplementary Material
