# Supplementary material for: JAK2V617F variant allele frequency >50% identifies patients with polycythemia vera at high risk for venous thrombosis
Source: Blood Cancer J. 2021 Dec 11;11(12):199. doi: 10.1038/s41408-021-00581-6 (PMC8665926; doi:10.1038/s41408-021-00581-6)
Supplement: Supplementary file 1 — Supplementary Material [file 41408_2021_581_MOESM1_ESM.pdf]

Supplementary Material

<u>Supplement to</u>: ***JAK2*V617F variant allele frequency >50% identifies patients with polycythemia vera at high risk for venous thrombosis**

Supplementary material legend (pages 2-5):

1. **Supplemental Figure 1.** ROC curve for *JAK2*V617F VAF as a risk variable for venous thrombosis using PV patients from training cohort.
2. **Supplemental Figure 2.** Myelofibrosis-free (A), leukemia-free (B), and overall (C) survival Kaplan Meier curves in PV patients from training cohort stratified by their VAF (>/≤50%).
3. **Supplemental Table 1.** Type of thrombotic events occurring at diagnosis or during follow-up in validation cohort.

Supplemental Figure 1

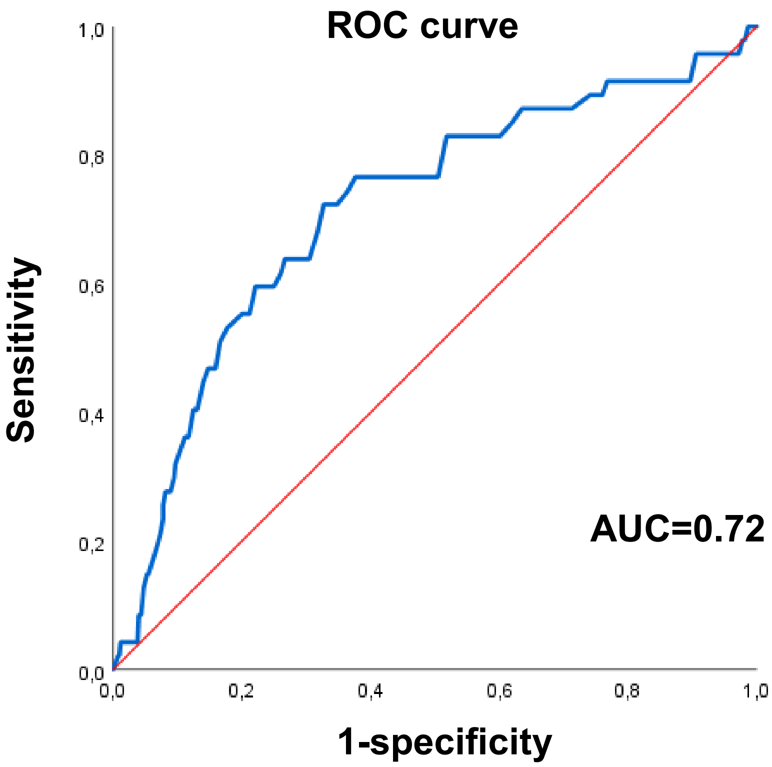

Supplemental Figure 2

A

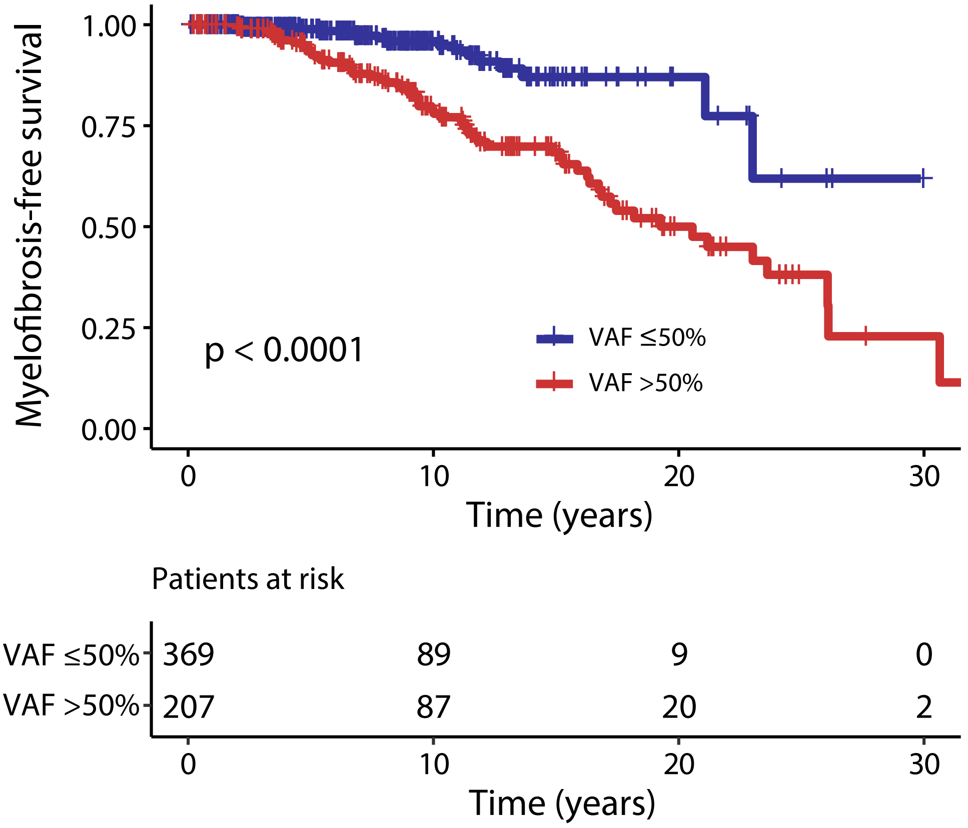

B

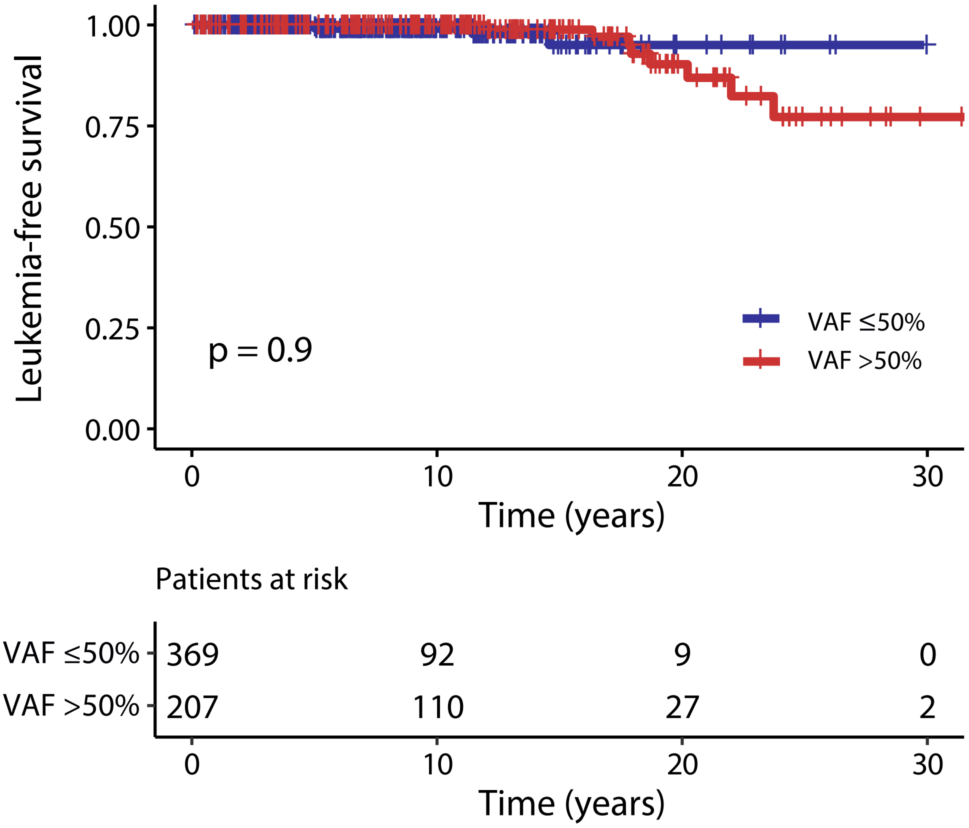

c

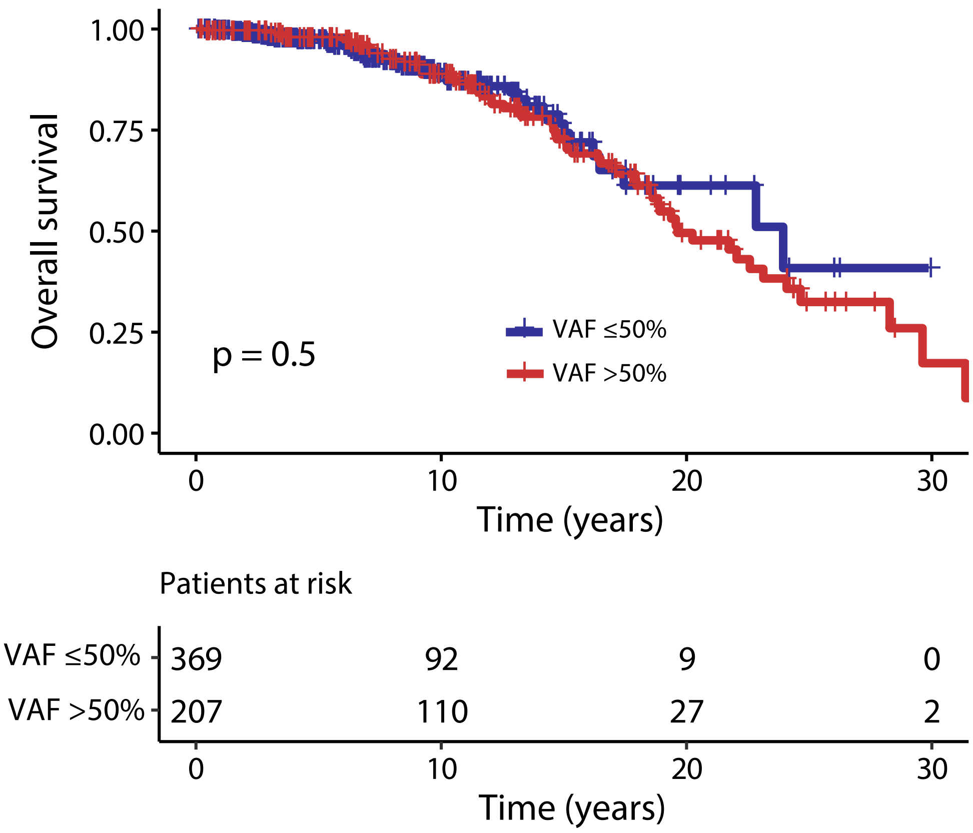
Overall survival
1.00
0.75
0.50
0.25
0.00
p = 0.5
VAF ≤50%
VAF >50%
0
10
20
30
Time (years)
Patients at risk
VAF ≤50%
VAF >50%
369
92
9
0
207
110
27
2
0
10
20
30
Time (years)

Supplemental Table 1. Type of thrombotic events occurring at diagnosis or during follow-up in the validation cohort.

**Catholic University-Policlinico Gemelli (n=289)**		
	**At diagnosis**	**During follow-up**
**Arterial thrombosis** (n, %)	n=17	n=45
Ischemic Stroke	6 (28.9)	12 (26.7)
Transient ischemic attack	5 (22.4)	14 (31.1)
Acute myocardial infarction	4 (34.2)	10 (22.2)
Unstable angina	1 (5.3)	2 (4.4)
Ischemic stroke + splenic infarction	1 (6.6)	--
Splenic infarction	--	2 (4.4)
Peripheral thrombosis	--	4 (9.0)
Retinal thrombosis	--	1 (2.2)
**Venous thrombosis** (n, %)	n=9	n=43
Deep vein thrombosis	4 (44.5)	14 (32.5)
Pulmonary embolism + deep vein thrombosis	2 (22.2)	4 (9.3)
Cerebral vein thrombosis	3 (33.3)	2 (4.7)
Pulmonary embolism	--	3 (7)
Superficial vein thrombosis	--	20 (46.5)
